# Analytical Model for Springback Prediction of CuZn20 Foil Considering Size Effects: Weakening versus Strengthening

**DOI:** 10.3390/ma13214929

**Published:** 2020-11-02

**Authors:** Xin Guan, Zhenwu Ma, Chunju Wang, Haidong He, Yuanjing Zhang, Xinwei Wang, Weiwei Zhang

**Affiliations:** 1 College of Mechanical Engineering, Suzhou University of Science and Technology, Suzhou 215009, China; guanxinxin9999@163.com (X.G.); mazw@usts.edu.cn (Z.M.); zhangyj@usts.edu.cn (Y.Z.); 2 School of Mechanical and Electrical Engineering, Robotics and Microsystems Center, Soochow University, Suzhou 215131, China; 3 Laboratory for Space Environment and Physical Sciences, Harbin Institute of Technology, Harbin 150080, China; wxw8326@163.com; 4 Institute of Electronic Engineering, China Academy of Engineering Physics, Mianyang 621999, China; zhangweiwei0509103@163.com

**Keywords:** springback prediction, analytical model, size effects, surface grain, strain gradient

## Abstract

The prediction of springback angle for ultra-thin metallic sheets becomes extremely difficult with the existence of size effects. In this study, size effects on the springback behavior of CuZn20 foils are investigated by experiments and analytical methods. The experimental results reveal that the springback angle first decreases gradually and then increases markedly with the decrease of foil thickness, which cannot be analyzed by current theoretical models. Then, an analytical model based on the Taylor-based nonlocal theory of plasticity is developed, in which the drastic increases of both the proportion of surface grains and the strain gradient are taken into account. Moreover, the influence of strain gradient is modified by the grain-boundary blocking factor. The calculation results show that the springback angle of foils is determined by the intrinsic competition between the decrement angle caused by surface grains and the increment angle caused by the strain gradient. Besides, the relative error of predicted springback angle by the model is less than 15%, which means that the developed model is very useful for improving the quality of micro sheet parts with high accuracy of springback prediction.

## 1. Introduction

Nowadays, multiple fields such as medical equipment, aircraft, and micro electro mechanical systems show growing trends in miniaturization and lightweight, which leads to a big increase in the demand for micro-bending parts [1,2]. For most of the micro-bending parts, in particular, heart stent, corrugated sheet, lead frames, and so on, the final shapes have an important influence on their functions [3,4]. However, because of complicated size effects, the springback behavior of foils is difficult to predict, which leads to the lack of theoretical support for designing the foil bending process.

At present, research on the size effects on foil forming can be roughly divided into two fields. On one hand, some researchers focus on the weakening material deformation behavior caused by surface grains. The surface grain theory holds that, compared with internal grains, surface grains have less constraint owing to the presence of free surface, so the deformation of surface grains is much easier than that of internal grains [5]. In the micro-compression test of copper performed by Geiger et al., they found that the flow stress decreases with the specimen size [6]. Besides, it has been demonstrated that the flow stress decreases with the thickness of thin-sheet via the tensile test of pure copper [7]. Because of the small grain numbers in micro parts, it is necessary to consider the weakening effect of surface grains when analyzing the deformation behavior of micro-scale materials. On the other hand, the strengthening material deformation behavior caused by the strain gradient has become a research priority. The strain gradient theory holds that when the geometric non-uniform deformation of micro-scale materials occurs, the density of geometrically necessary dislocations increases rapidly. Geometrically necessary dislocations can “pin” somewhere in the form of dislocation barriers during deformation, causing the material strengthening [8]. In the micro-twisting test of thin copper wires, it is reported that the normalized shear strength increases with the wire diameter decreasing from 170 μm to 12 μm [9]. In the micro-bending test of thin nickel beams, Stolken et al. found that the normalized bending moment increases rapidly when the thickness of foils reduces from 100 μm to 12.5 μm [10]. Based on the correlation between geometrically necessary dislocations and the strain gradient, a series of strain gradient plasticity theories have been proposed [11,12,13,14]. Nix and Gao et al. subdivided the dislocations into statistically stored dislocations and geometrically necessary dislocations in the Taylor hardening relationship, which avoids the introduction of higher-order stresses [15]. Based on this, the Taylor-based nonlocal theory of plasticity (TNT theory) is developed by expressing the density of geometrically necessary dislocations as a nonlocal integral of the strain field [16]. Compared with other strain gradient theories, there are fewer model parameters in the TNT theory, contributing to its high convenience in predicting the geometric non-uniform deformation behavior of micro-scale materials [17].

Based on the above reviews of previous research work, it can be concluded that, as the thickness of foils decreases continuously, both surface grains and the strain gradient can affect the springback behavior of foils significantly. However, there is still a lack of in-depth research on the combined influence of surface grains and the strain gradient on the springback behavior of foils. Ma et al. proposed that, apart from the weakening effect of surface grains, the strengthening effect of strain gradient should also be considered when analyzing the bending behavior of foils, but no quantitative examination was performed [18]. Therefore, it is necessary to develop an analytical model with consideration of the influence of both surface grains and the strain gradient.

In this study, the size effects on the springback behavior of CuZn20 foils were quantitatively investigated with experimental and analytical methods. Then, an analytical model was established with overall consideration of the weakening effect of surface grains and the strengthening effect of strain gradient. Furthermore, factors that affect the springback behavior of foils were quantitatively analyzed with the developed analytical model.

## 2. Materials and Methods 

**Figure 1:**
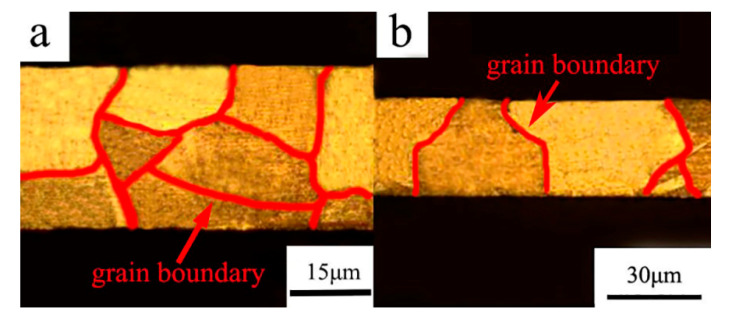
Typical metallographical micrographs of foils with a thickness of 50 μm (**a**) and 30 μm (**b**).

CuZn20 foils with a thickness from 30 μm to 400 μm were applied in this study. The physical property parameters of CuZn20 foils are shown in Table 1. After the annealing treatment, foils with similar grain sizes (around 35 μm) were selected for subsequent experiments and analysis (Table 2). As shown in Figure 1, typical metallographical micrographs of foil specimens demonstrated that the main difference between foils with different thickness is the number of grains in the foil thickness direction. The mechanical properties of foils were obtained via uniaxial tensile tests. The initial measuring length and width for all specimens were kept constant at 50 mm and 12.5 mm, respectively.

The foil bending experiment was designed based on the principle of similarity. As shown in Table 3, all parameters related to bending were adjusted according to scaling factor λ, which is proportional to the thickness of foils.

**Figure 2:**
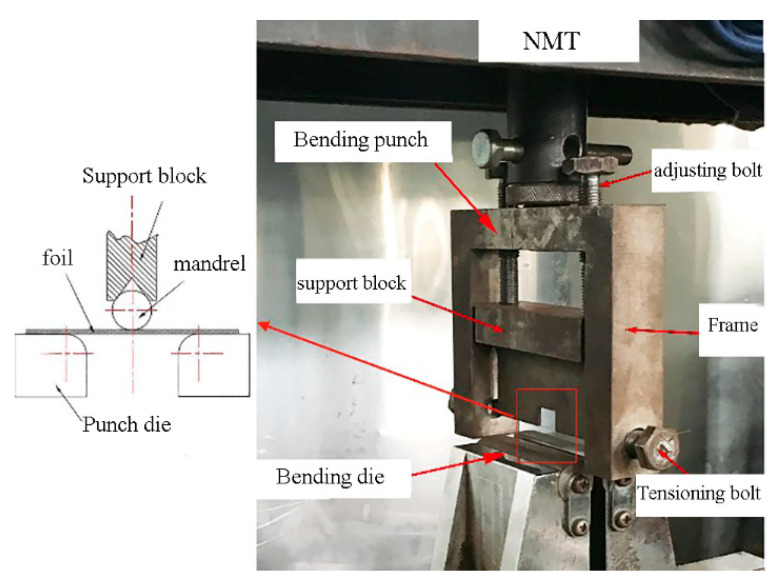
Experimental device of foil bend forming. NMT, material testing machine.

**Figure 3:**
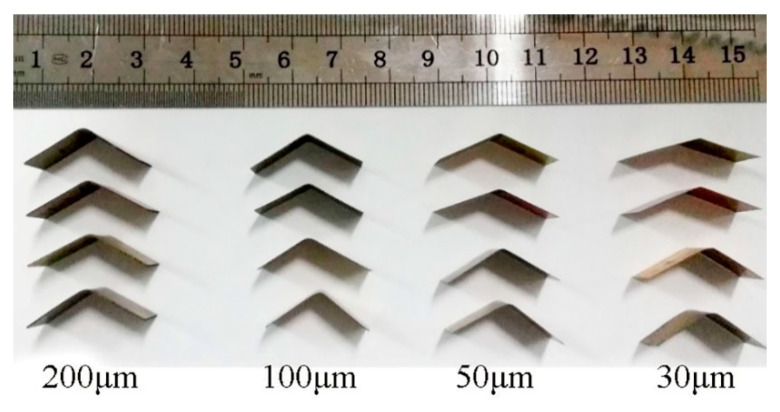
Morphology of foil specimens with different thickness after bending.

In order to ensure the experimental accuracy, a foil three-point bending device was designed. As shown in Figure 2, the device is connected to an electrical universal material testing machine (NMT) and the parameters are adjusted according to Table 3. The length and width of bending specimens are fixed at 25 mm and 10 mm, respectively, and the bending angle is 90°. After bending, foils were photographed and measured using the Photoshop software with an accuracy of 0.01° (Figure 3). The springback angle was obtained by averaging the data of six tests for each thickness group.

## 3. Results and Discussion

**Figure 4:**
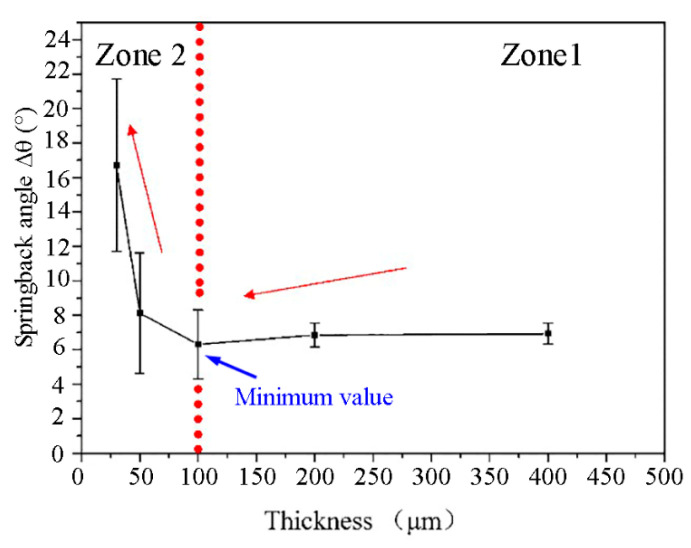
The variation of springback angle with thickness.

As shown in Figure 4, with the decrease of foil thickness from 400 μm to 30 μm, two contradictory springback trends were demonstrated in different thickness zones. In zone 1, as the foil thickness decreased from 400 μm to 200 μm, the springback angle decreased gradually from 6.93° to 6.65°. However, in zone 2, the springback angle increased significantly from 5.97° to 16.71° with the further decrease of foil thickness from 100 μm to 30 μm. The minimum value of springback angle occurred at the critical thickness of two zones (around 100 μm). Besides, it can be observed that the standard deviation of springback angle increased significantly with the decrease of foil thickness, indicating that the influence of individual grain heterogeneity (size, shape, orientation) on the springback behavior of foils enhanced markedly with the decrease of grain numbers in the thickness direction.

**Figure 5:**
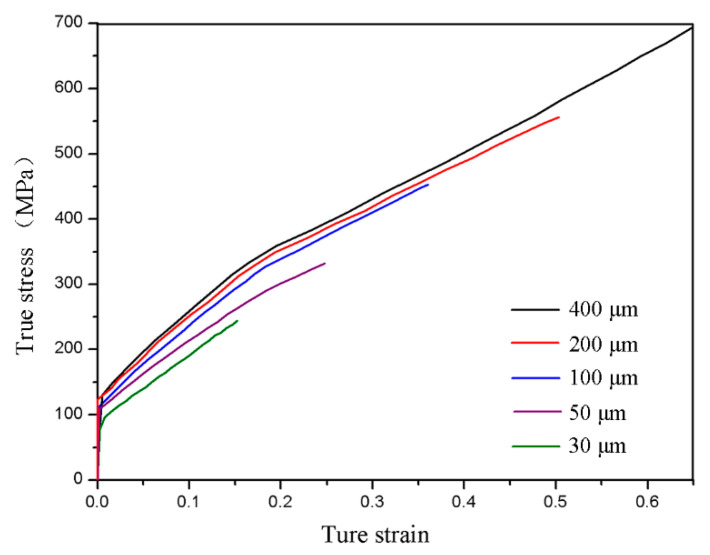
The flow stress of foils with different thickness.

According to the bending theory, lower flow stress can cause smaller springback angles. The flow stress curves of CuZn20 foils with different thickness are presented in Figure 5, indicating that the foil flow stress decreases with the thickness and can be illuminated by the surface grain theory. Because, with the decrease of foil thickness, the proportion of surface grains increases (Table 2), the continuously enhanced weakening effect of surface grains leads to a larger proportion of plastic deformation area in the foils during the bending process, resulting in the decrease of springback angle. However, the surface grain theory cannot explain the drastic increase of springback angle in zone 2.

When the foil thickness is near the intrinsic material length, which is estimated on the order of several microns, the influence of strain gradient increased markedly. According to the correlation between geometrically necessary dislocations and the strain gradient [13,14,15,16,17], the density of geometrically necessary dislocations $\rho_{g}$ in the foil bending can be calculated as follows:

(1) $$\rho_{g}=\frac{\eta }{b}$$

where η is the effective strain gradient, and b is the modulus of Burgers vector.

On the other hand, the scalar expression of the strain gradient η along the thickness direction is as follows:

(2) $$\eta =\frac{\varepsilon_{s}-\varepsilon_{0}}{t/2}=\frac{\varepsilon_{s}-0}{t/2}=\frac{1}{R}=k$$

where ε_s_ is the surface strain, ε_0_ = 0 is the strain of the bending neutral layer, R is the radius of the bending neutral layer, t is the foil thickness, and k is the bending curvature.

In the similarity bending experiment of CuZn20 foils, the surface strain of all foils is the same. From Equations (1) and (2), it can be seen that, when the foil thickness is small, the strain gradient along the thickness direction is large. Therefore, the hardening ability of the material will increase dramatically with the increase of the density of geometrically necessary dislocations.

Based on the above analyses, it can be concluded that the weakening effect of surface grains and the strengthening effect of strain gradient both increase with the decrease of foil thickness. However, the reason for the two contradictory springback trends in different thickness zones (Figure 4) is still unclear. In the following, a quantitative analysis will be performed.

## 4. Analytical Model

### 4.1. Expression of Strain Gradient as a Nonlocal Integral of Strain

Similar to the expression of equivalent strain in classical theories of plasticity, Gao et al. established the tensor expression of equivalent strain gradient as follows [19]:

(3) $$\eta =\sqrt{\frac{1}{4}\eta_{\mathrm{ijk}}^{\prime }\eta_{\mathrm{ijk}}^{\prime }}$$

where $\eta_{\mathrm{ijk}}^{\prime }$ is a third-order deviatoric strain gradient tensor. 

Based on the influence mechanism of geometrically necessary dislocations on the geometric non-uniform deformation behavior of micro-scale materials, the strain gradient is solved under the non-local plastic theory framework. The strain gradient η is expressed as the nonlocal integration of strain in a micro-cubic cell [19] and the third-order deviatoric strain gradient tensor $\eta_{\mathrm{ijk}}^{\prime }$ in Equation (3) can be expressed as follows [16]:

(4) $$\eta_{\mathrm{ijk}}^{\prime }=\frac{1}{I_{\epsilon }}\int_{V_{\mathrm{cell}}}[\epsilon_{\mathrm{ik}}\left(x\right)\xi_{j}+\epsilon_{\mathrm{jk}}\left(x\right)\xi_{i}-\epsilon_{\mathrm{ij}}\left(x\right)\xi_{k}-\frac{1}{4}\left(\delta_{\mathrm{ik}}\xi_{j}+\delta_{\mathrm{jk}}\xi_{i}\right)\epsilon_{\mathrm{pp}}]\mathrm{dV}$$

where ξ is an integral term, $V_{\mathrm{cell}}$ is the volume of the micro-cubic cell, and $I_{\epsilon }$ is the moment of inertia of the micro-cubic cell.

### 4.2. Material Hardening Behavior under Strain Gradient

It is assumed that, under the condition of geometric uniform deformation, the hardening behavior of the material follows the power exponential hardening relationship:

(5) $$\sigma_{\mathrm{cla}}=\sigma_{\mathrm{ref}}f\left(\epsilon \right)=\sigma_{\mathrm{ref}}\epsilon^{n}$$

where $\sigma_{\mathrm{ref}}$ is the reference stress and n is the strain hardening exponent.

Based on the Taylor hardening model, Gao et al. deduced the expression of the influence of strain gradient [19]:

(6) $$f_{\mathrm{sg}}\left(\eta \right)=\iota \eta =18\zeta^{2}{\left(\frac{G}{\sigma_{\mathrm{ref}}}\right)}^{2}b\eta$$

where $\iota =18\zeta^{2}{\left(\frac{G}{\sigma_{l}}\right)}^{2}b$ is the intrinsic material length, ζ is an empirical constant on the order of 1, G and b are the physical property parameters of materials, and $\sigma_{\mathrm{ref}}$ is the mechanical property parameters of materials.

Then, a stress balance relationship expression is developed as follows:

(7) $$\sigma_{c}=\sigma_{\mathrm{ref}}{\left(f_{s}^{\beta }\left(\epsilon \right)+f_{\mathrm{sg}}^{\beta }\left(\eta \right)\right)}^{\frac{1}{\beta }}$$

where $\sigma_{c}$ is the combined stress, $f_{s}^{\beta }\left(\epsilon \right)$ is the expression of the influence of strain, $f_{\mathrm{sg}}^{\beta }\left(\eta \right)$ is the expression of the influence of strain gradient, and β is the adjustment factor.

**Figure 6:**
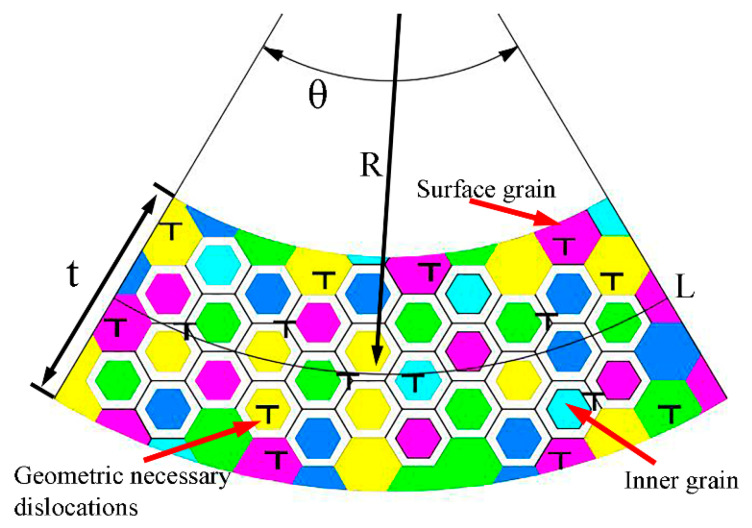
Schematic diagram of the distribution of geometrically necessary dislocations in foil bending.

In order to maintain the dimensional balance of the effect of strain gradient with the traditional strain hardening, the intrinsic material length term ι is introduced into the strain gradient model [16]. However, during the application process, the calculated values of intrinsic material length vary greatly. Besides, it has been reported that the intrinsic material length of annealed copper is more than twice that of cold hardening copper [15,16]. In other words, the intrinsic material length varies with the grain size [10,20]. It is known that the grain is composed of the grain-interior region and the grain-boundary region [21]. During the deformation process, the grain-boundary region and geometrically necessary dislocations both play important roles of coordination. Moreover, the atom arrangement in the grain-boundary region is disordered with dislocations defects. Therefore, when geometrically necessary dislocations are located in the grain-boundary region, their effect will be blocked. There are two kinds of grains in foils, surface grains and internal grains. Surface grains are not constrained by the grain-boundary region. Therefore, the effect of geometrically necessary dislocations will be blocked only when they are located in the grain-boundary region of internal grains (Figure 6). In order to accurately characterize the blocking effect of grain-boundary region on geometrically necessary dislocations, a method is developed as follows.

As shown in Figure 6, the grain shape is assumed to be regular hexagon, and the proportion of grain-boundary region in the entire foil cross-section (two-dimensional) $P_{gb}$ is expressed as follows:

(8) $$P_{gb}=\frac{(L-2d)(t-2d)\left[1-{\left(\frac{d-2T}{d}\right)}^{2}\right]}{Lt}=\frac{(t-2d)\left[1-{\left(\frac{d-2T}{d}\right)}^{2}\right]}{t}$$

where θ is the bend angle, L = Rθ is the length of the neutral layer, d is the grain size, t is the foil thickness, and T = 0.133d^0.7^ is the thickness of the grain-boundary region [21].

The grain-boundary blocking factor ω is defined as follows:

(9) $$\omega =\left(1-P_{gb}\right)$$

Equation (6) is modified as follows:

(10) $$f_{\mathrm{sg}}\left(\eta \right)=\omega \iota \eta =\left(1-P_{gb}\right)18\zeta^{2}{\left(\frac{G}{\sigma_{l}}\right)}^{2}b\eta$$

**Figure 7:**
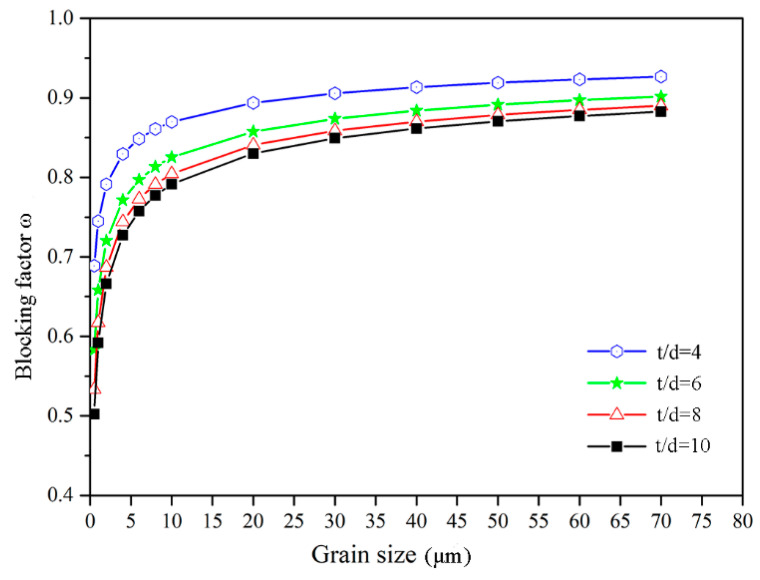
The variation of blocking factor ω with the grain size.

The change of blocking factor ω with the grain size is shown in Figure 7. It can be seen that the value of grain-boundary blocking factor ω decreases with the decrease of grain size, which can be interpreted as the increased thickness of grain-boundary region with the decrease of grain size. Besides, the blocking factor ω is decreased with the increase of t/d value, which is caused by the decreased percentage of surface grains with the increase of t/d value.

### 4.3. Mechanical Analysis of Foil Bend Forming

#### 4.3.1. Equivalent Strain and Equivalent Strain Gradient

**Figure 8:**
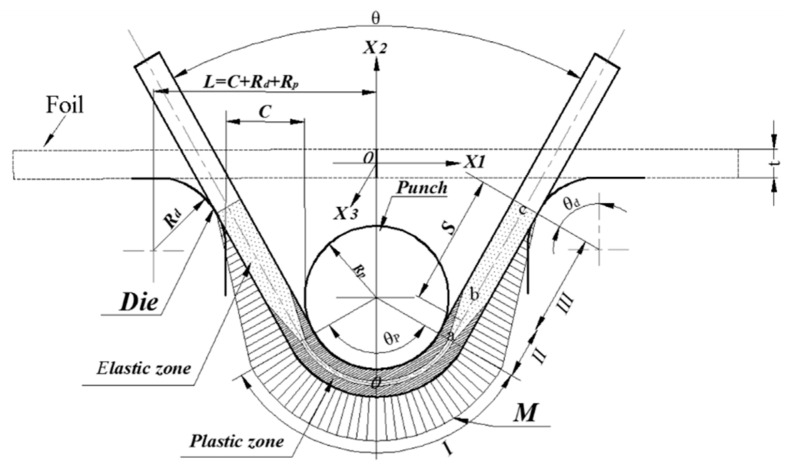
Schematic of the coordinate system and the bending moment distribution.

The coordinate system in the foil was established, as shown in Figure 8. X1 is along the foil neutral axis, X2 is along the foil thickness direction, and X3 is along the foil width direction. According to the principle of plane strain deformation ($\epsilon_{33}=0$) and incompressibility ($\epsilon_{\mathrm{kk}}=0$) in foil bend forming, the strain can be expressed as follows:

(11) $$\epsilon_{11}=-\epsilon_{22}=kx_{2},\epsilon_{12}=0$$

Using the calculation method of third-order deviatoric strain gradient tensor $\eta_{\mathrm{ijk}}^{\prime }$ in Equation (4), $\eta_{112}^{\prime }$ can be calculated as follows:

(12) $$\eta_{112}^{\prime }=\frac{1}{I_{\epsilon }}\int_{V_{\mathrm{cell}}}\left[-\epsilon_{11}\left(x\right)\xi_{2}\right]\mathrm{dV}=\frac{1}{I_{\epsilon }}\int_{V_{\mathrm{cell}}}\left[-k\left(x_{2}+\xi_{2}\right)\xi_{2}\right]\mathrm{dV}=-k$$

The other nonzero components of strain gradients can be obtained with a similar method:

(13) $$\eta_{112}^{\prime }=\eta_{222}^{\prime }=-k,\;\eta_{121}^{\prime }=\eta_{211}^{\prime }=k$$

In addition, according to the classical forming theory, the equivalent strain can be expressed as follows:

(14) $$\epsilon =\frac{2}{\sqrt{3}}k\left|x_{2}\right|$$

Therefore, the equivalent strain gradient in foil bend forming can be obtained based on Equation (3):

(15) η=k

#### 4.3.2. Stress Analysis

The constitutive equations for the deformation theory of TNT are as follows: $\sigma_{kk}=3K\epsilon_{kk}$

(16) $$\sigma_{ij}{}^{\prime }=\frac{2\sigma_{ref}\sqrt{f^{2}\left(\epsilon \right)+\iota \eta }}{3\epsilon }\epsilon_{ij}{}^{\prime }$$

where K = E/[3(1 − 2γ)] is the elastic bulk modulus, E is the Young’s modulus, and γ is the Poisson’s ratio.

Then, the nonvanishing deviatoric stresses in foil bend forming can be calculated as follows:

(17) $$\sigma_{11}{}^{\prime }=-\sigma_{22}{}^{\prime }=\mathrm{sign}\left(x_{2}\right)\frac{\sigma_{c}}{\sqrt{3}}$$

where $\sigma_{c}$ is the material hardening behavior under the strain gradient described in Equation (7), and $\mathrm{sign}\left(x_{2}\right)$ stands for the sign of $x_{2}$.

Furthermore, the hydrostatic stress is expressed by considering the traction-free boundary conditions on the top and bottom surfaces of foils:

(18) $$\sigma_{\mathrm{kk}}=-3\sigma_{22}{}^{\prime }$$

On these bases, the traction at the cross section of foils is as follows:

(19) $$\sigma_{11}=\sigma_{11}{}^{\prime }+\frac{1}{3}\sigma_{\mathrm{kk}}=\mathrm{sign}\left(x_{2}\right)\frac{2\sigma_{c}}{\sqrt{3}}$$

#### 4.3.3. Bending Moment Calculation 

It is assumed that the stress at point $x_{2}=mt/2$ (0<m<1) in the thickness direction of foils reaches the yield stress of the material. Then, the deformation zone along the thickness direction can be divided into the elastic deformation zone and plastic deformation zone. The width of the foil is W. Therefore, the bending moment M can be expressed as the sum of the elastic bending moment $M_{e}$ and the plastic bending moment $M_{P}$:

$$M=M_{e}+M_{P}=$$

(20) $$\int_{-\frac{t}{2}}^{\frac{t}{2}}\sigma_{1}\left(x_{2}\right)x_{2}Wdx_{2}=\int_{-m\frac{t}{2}}^{m\frac{t}{2}}\sigma_{1}\left(x_{2}\right)x_{2}Wdx_{2}+2\int_{m\frac{t}{2}}^{\frac{t}{2}}\sigma_{1}\left(x_{2}\right)x_{2}Wdx_{2}$$

In the elastic deformation zone, the stress–strain relationship follows Hooke’s law. The elastic bending moment $M_{e}$ can be expressed as follows:

(21) $$M_{e}=\int_{-m\frac{t}{2}}^{m\frac{t}{2}}\sigma_{1}\left(x_{2}\right)x_{2}Wdx_{2}=\frac{WE^{\prime }km^{3}t^{3}}{12}\;-m\frac{t}{2}<x_{2}<m\frac{t}{2}$$

where $E^{\prime }=E/\left(1-\gamma^{2}\right)$ and k is the bending curvature.

The plastic bending moment $M_{P}$ can be expressed as follows:

(22) $$M_{P}=2\int_{m\frac{t}{2}}^{\frac{t}{2}}\sigma_{1}\left(x_{2}\right)x_{2}Wdx_{2}=2W\int_{m\frac{t}{2}}^{\frac{t}{2}}x_{2}\frac{2\sigma_{c}}{\sqrt{3}}dx_{2}\;m\frac{t}{2}<x_{2}<\frac{t}{2}$$

Then, put Equation (7) into Equation (22), wherein the values of $\sigma_{\mathrm{ref}}$ and n can be obtained by fitting the tensile test data of foils, and β = 1 is determined by fitting the foils’ bending experimental results (detailed methods are described in [11]), thus

(23) $$\begin{array}{l} M_{P}=\frac{4W}{\sqrt{3}}\int_{m\frac{t}{2}}^{\frac{t}{2}}x_{2}\sigma_{\mathrm{ref}}\left(\epsilon^{n}+\omega \iota \eta \right)dx_{2}=\frac{4W\sigma_{\mathrm{ref}}}{\sqrt{3}}\int_{m\frac{t}{2}}^{\frac{t}{2}}\left[x_{2}{\left(\frac{2}{\sqrt{3}}kx_{2}\right)}^{n}+\omega \iota kx_{2}\right]dx_{2}\\ \;=\frac{4{W\sigma }_{\mathrm{ref}}{\left(2k\right)}^{n}}{\left(n+2\right){\sqrt{3}}^{n+1}}\left[{\left(\frac{t}{2}\right)}^{n+2}-{\left(m\frac{t}{2}\right)}^{n+2}\right]+\frac{\omega \iota kW\sigma_{ref}}{2\sqrt{3}}\left(t^{2}-m^{2}t^{2}\right)\\ \;=M_{Ps}+M_{Psg} \end{array}$$

where ι is the intrinsic material length, $M_{Ps}$ is the part of plastic bending moment related to the equivalent strain (statistical storage dislocations), and M_*psg*_ is the part of the plastic bending moment related to the equivalent strain gradient (geometrically necessary dislocations).

The yield stress $\sigma_{s}$ of foils with different thickness dimensions can be obtained by the tensile test. The value of m is calculated as follows:

(24) $$\sigma_{s}=\sigma_{\mathrm{ref}}\epsilon^{n}=\sigma_{\mathrm{ref}}{\left(\frac{2}{\sqrt{3}}kx_{2}\right)}^{n}=\sigma_{\mathrm{ref}}{\left(\frac{tkm}{\sqrt{3}}\right)}^{n}$$

(25) $$m={\left(\frac{\sigma_{s}}{\sigma_{\mathrm{ref}}}\right)}^{\frac{1}{n}}\frac{\sqrt{3}}{\mathrm{tk}}$$

#### 4.3.4. Springback Calculation

As shown in Figure 8, O is the vertex of the punch, a is the tangent point of the punch and the foil, b is the transition point of the elastic deformation and plastic deformation in the longitudinal direction of foils, and c is the tangent point of the foil and the die. According to [22], the following assumptions are made on the bending moment distribution in the foil deformation zone: (i) the arc from point o to a is the elastic-plastic bending, wherein the section from $-\frac{mt}{2}$ to $\frac{mt}{2}$ in the thickness direction is a pure elastic bending and the section from $\frac{mt}{2}$ to $\frac{t}{2}$ in the thickness direction is a pure plastic bending; (ii) the line from point a to b is the elastic-plastic bending deformation, and the line from point b to c is the pure elastic bending deformation; and (iii ) the bending moment from point c to a follows a linear distribution. Therefore, by calculating the length of the bending line from point c, the bending moment can be expressed as follows:

(26) $$\{\begin{array}{c} M=M_{P}+M_{e},\;x\;\text{is in the arc ao}\\ M=\frac{L_{cx}}{L_{ca}}(M_{P}+M_{e}),\;\text{x is in the line ca} \end{array}$$

The springback angle dθ along the longitudinal micro-arc ds is calculated as follows:

(27) $$d\theta =\frac{\mathrm{ds}}{∆R}=\mathrm{ds}∆k=\frac{M\mathrm{ds}}{E^{\prime }I}$$

where $I=\frac{Wt^{3}}{12}$ is the moment of inertia of the foil.

The springback angle ∆θ of the foil can be expressed by integrating ds along the bending line:

(28) $$∆\theta =2\left(\int_{0}^{L_{ca}}\frac{L_{cx}}{L_{ca}}\frac{\left(M_{P}+M_{e}\right)}{E^{\prime }I}\mathrm{ds}+\int_{S_{a}}^{S_{0}}\frac{M_{P}+M_{e}}{E^{\prime }I}\mathrm{ds}\right)=\frac{M_{P}+M_{e}}{E^{\prime }I}(L_{ca}+2\hat{S_{\mathrm{ao}}})$$

where $L_{ca}$ is the length of line ca and $\hat{S_{\mathrm{ao}}}$ is the length of arc ao.

$L_{ca}$ can be obtained according to the bending geometric relationship:

(29) $$L_{ca}=\left[L-\left(R_{d}+\frac{t}{2}\right)\sin\theta_{d}-\left(R_{P}+\frac{t}{2}\right)\sin\left(\frac{\theta_{P}}{2}\right)\right]/\cos\left(\frac{\theta }{2}\right)$$

where $\theta_{d}$, $\theta_{P},\;\mathrm{and}\;\theta$ are the die-foil contact angle, punch-foil contact angle, and bend angle, respectively.

$\hat{S_{\mathrm{ao}}}$ can be calculated as follows:

(30) $$\hat{S_{\mathrm{ao}}}=\left(R_{P}+\frac{t}{2}\right)\cdot \frac{\theta_{P}}{2}$$

Assuming that $\theta_{d}=\frac{\theta_{P}}{2}=\frac{\theta }{2}$, Equation (26) can be expressed as follows:

(31) $$\begin{array}{l} ∆\theta =\frac{M_{P}+M_{e}}{E^{\prime }I}(L_{ca}+2\hat{S_{\mathrm{ao}}})=\frac{M_{Ps}+M_{Psg}+M_{e}}{E^{\prime }I}(L_{ca}+2\hat{S_{\mathrm{ao}}})\\ \;=\left[\frac{12\sigma_{\mathrm{ref}}k^{n}t^{n}(1-m^{n+2})\left(\frac{30}{\sqrt{2}}-11+\frac{11\pi }{4}\right)}{\left(n+2\right){\sqrt{3}}^{n+1}E^{\prime }}+{\mathrm{km}}^{3}\left(\frac{30}{\sqrt{2}}-11+\frac{11\pi }{4}\right)\right]\\ \;+\frac{6{\omega \iota k\sigma }_{\mathrm{ref}}\left(1-m^{2}\right)\left(\frac{30}{\sqrt{2}}-11+\frac{11\pi }{4}\right)}{\sqrt{3}E^{\prime }}=∆\theta_{\mathrm{cla}}+∆\theta_{\mathrm{sg}} \end{array}$$

where $∆\theta_{\mathrm{cla}}=\frac{12\sigma_{\mathrm{ref}}k^{n}t^{n}(1-m^{n+2})\left(\frac{30}{\sqrt{2}}-11+\frac{11\pi }{4}\right)}{\left(n+2\right){\sqrt{3}}^{n+1}E^{\prime }}+{\mathrm{km}}^{3}\left(\frac{30}{\sqrt{2}}-11+\frac{11\pi }{4}\right)$ is the springback angle calculated with classical bending theory, and $∆\theta_{\mathrm{sg}}=\frac{6{\omega \iota k\sigma }_{\mathrm{ref}}\left(1-m^{2}\right)\left(\frac{30}{\sqrt{2}}-11+\frac{11\pi }{4}\right)}{\sqrt{3}E^{\prime }}$ is the springback angle caused by the strain gradient.

### 4.4. Application and Discussion

Transforming Equation (31) according to different theories, the springback angle of CuZn20 foils can be calculated from different perspectives:

(1) Classical bend forming theory (calculation with $∆\theta_{\mathrm{cla}}$, the mechanical properties of foils are represented by that of the 400 μm thick foils);
(2) Surface grain theory (calculation with $∆\theta_{\mathrm{cla}}$, the mechanical properties of foils are shown in Figure 5);
(3) Strain gradient theory (calculation with $∆\theta_{\mathrm{cla}}+∆\theta_{\mathrm{sg}}$, ω = 1 in $∆\theta_{\mathrm{sg}}$);
(4) The analytical model (calculation with $∆\theta_{\mathrm{cla}}+∆\theta_{\mathrm{sg}}$, ω is calculated as Equation (9) in $∆\theta_{\mathrm{sg}}$).

**Figure 9:**
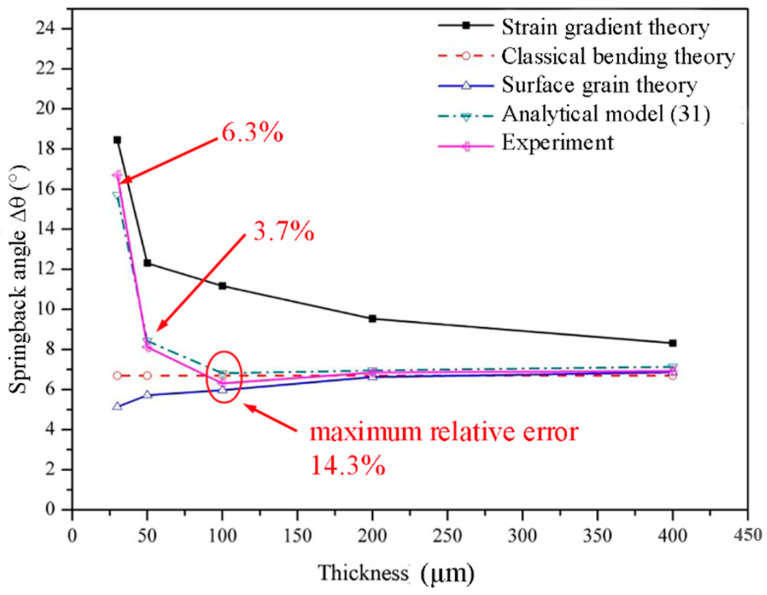
Comparison of calculation values by four methods and experimental results.

The comparison between calculated values and experimental results is shown in Figure 9. In the classical bending forming theory, the flow stress of foils is not affected by the thickness. In method (1), the weakening effect of surface grains and the strengthening effect of strain gradient in foil bending are not considered. Therefore, the mechanical properties of all foils are expressed as that of the 400 μm thick foils, so the calculated springback values of all foils are the same, resulting in significant deviation with the experimental results. Method (2) calculates the springback by taking advantage of the mechanical properties of foils obtained in experiments. Because the weakening effect of surface grains is considered, the calculated springback values decreases with the decrease of foil thickness. Therefore, the calculated value is close to the experimental results in zone 1, but deviates greatly in zone 2. In method (3), springback angles are calculated by combining the mechanical properties of foils with the strain gradient theory. Because of the consideration of deformation strengthening caused by the increased geometrically necessary dislocation density on the basis of the weakening effect of surface grains, the increased springback values as the foil thickness decreases in zone 2 can be characterized. However, as the CuZn20 foil is a polycrystalline material, the blocking effect of the grain-boundary region on geometrically necessary dislocations is not included in method (3), leading to larger analytical values than experimental results. In method (4), owing to the comprehensive consideration of the weakening effect of surface grains, the strengthening effect of strain gradient, and the blocking effect of the grain-boundary region on geometrically necessary dislocations in foil bend forming, the predicted springback values for CuZn20 foils agree well with the experimental results. As shown in Figure 9, the maximum relative error (14.3%) of the predicted springback angle by the model occurs at the critical thickness, and the average relative error of the model is less than 15%.

**Figure 10:**
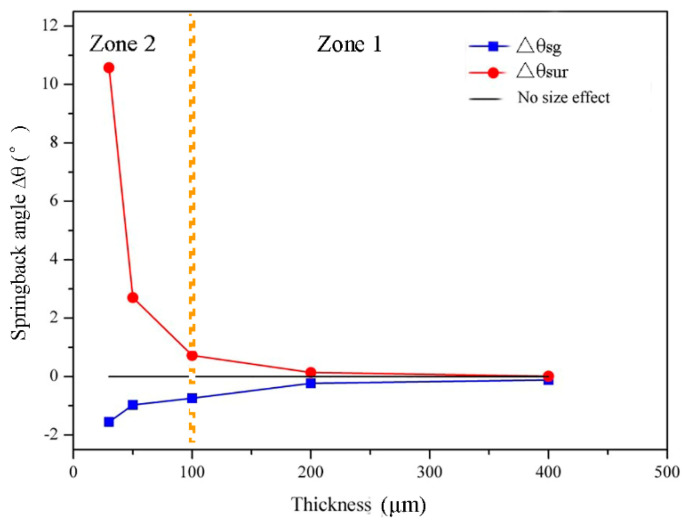
The variation of ∆θ_sur_ and ∆θ_sg_ with foil thickness.

The difference between springback angles calculated with the classical bend forming theory and the surface grain theory is the decrement angle $∆\theta_{\mathrm{sur}}$ caused by surface grains. The difference between springback angles calculated with the analytical model (method 4) and the surface grain theory is the increment angle $∆\theta_{\mathrm{sg}}$ caused by the strain gradient. The changes of $∆\theta_{\mathrm{sur}}$ and $∆\theta_{\mathrm{sg}}$ with foil thickness are shown in Figure 10. In general, as the thickness of foils decreases, the value of $∆\theta_{\mathrm{sur}}$ decreases, but the value of $∆\theta_{\mathrm{sg}}$ increases, both with growing rangeability. Then, referring to Figure 4, a partition is carried out on Figure 10 at the thickness of 100 μm. It can be seen that, in zone 1, the value of $∆\theta_{\mathrm{sg}}$ is small, so the springback angle is mainly dominated by $∆\theta_{\mathrm{sur}}$, leading to the decreasing springback angle with the decrease of foil thickness. When the foil thickness is 100 μm, the values of $∆\theta_{\mathrm{sur}}$ and $∆\theta_{\mathrm{sg}}$ are nearly equal, contributing to the smallest springback angle of foils. In zone 2, $∆\theta_{\mathrm{sg}}$ increases sharply as the foil thickness decreases, while $∆\theta_{\mathrm{sur}}$ decreases slowly. Therefore, $∆\theta_{\mathrm{sg}}$ dominates the material springback behavior, resulting in a marked increase in the springback angle of foils. This is the reason that two contradictory springback trends occur in different thickness zones (Figure 4).

## 5. Conclusions

In this study, size effects on the springback behavior of CuZn20 foils were quantitatively investigated with experimental and analytical methods. The following conclusions are obtained:

(1) With the decrease of the foil thickness, the springback of foils shows two contradictory trends that are divided by a critical thickness, and the springback angle is the minimum at the critical thickness.
(2) An analytical model based on Taylor-based nonlocal theory of plasticity is developed, in which the drastic increases of both the proportion of surface grains and the strain gradient are taken into account. Moreover, the influence of strain gradient in the model is modified by considering the blocking effect of the grain-boundary region on geometrically necessary dislocations.
(3) The springback angle of foils is jointly determined by the decrement angle caused by surface grains and the increment angle caused by the strain gradient. The appearance of springback trend is ultimately determined by the intrinsic competition between the weakening and strengthening contributions resulting from size effects.
(4) The relative error of the predicted springback angle by the model is less than 15%.

## Figures and Tables

**Table 1:** Physical property parameters of CuZn20 foils.

Young’s Modulus E	Shear Modulus G	Burger’s Vector b	Poisson’s Ratio γ
91 GPa	34.2 GPa	3.6 × 10 ^−7^ mm	0.33

**Table 2:** Material parameters of experimental CuZn20 foils.

Thickness (t, μm)	Annealing Conditions	Grain Size (d, μm)	P_s_
30	600 °C, 1 h	34.2 ± 2.4	100%
50	500 °C, 1 h	35.3 ± 2.7	100%
100	500 °C, 1 h	33.8 ± 1.9	66.7%
200	500 °C, 1.5 h	36.2 ± 3.1	36.3%
400	400 °C, 1 h	34.6 ± 2.3	17.2%

P_s_ = 2d/t is the proportion of surface grains.

**Table 3:** Parameters in similarity bending experiments.

Thickness t (μm)	Scaling Factor λ	Mandrel Diameter D_d_ (mm)	Die Diameter D_p_ (mm)	Clearance between Mandrel and Die C (mm)	Punch Speed v (mm/min)
30	0.3	0.3	0.3	0.15	0.3
50	0.5	0.5	0.5	0.25	0.5
100	1	1	1	0.5	1
200	2	2	2	1	2
400	4	4	4	2	4
